# Considering inequities in national dementia strategies: breadth, depth, and scope

**DOI:** 10.1186/s12939-024-02166-8

**Published:** 2024-04-16

**Authors:** Claire Godard-Sebillotte, Sanjna Navani, Georgia Hacker, Isabelle Vedel

**Affiliations:** 1 The Research Institute of the McGill University Health Centre, Montreal, QC Canada; 2 Department of Medicine, Division of Geriatrics, McGill University, Montreal, QC Canada; 3 The Michael G DeGroote School of Medicine, McMaster University, Hamilton, ON Canada; 4 Lady Davis Institute, Jewish General Hospital, Montreal, QC Canada; 5 Department of Family Medicine, McGill University, Montreal, QC Canada

**Keywords:** Dementia, National dementia strategies, OECD countries, Social determinants of health, Health inequities, Objectives

## Abstract

**Background:**

Considering that dementia is an international public health priority, several countries have developed national dementia strategies outlining initiatives to address challenges posed by the disease. These strategies aim to improve the care, support, and resources available to meet the needs of persons living with dementia and their care partners and communities. Despite the known impact of social determinants of health on dementia risk, care, and outcomes, it is unclear whether dementia strategies adequately address related inequities. This study aimed to describe whether and how national dementia strategies considered inequities associated with social determinants of health.

**Methods:**

We conducted an environmental scan of the national dementia strategies of countries that are part of the Organisation for Economic Cooperation and Development (OECD). Included strategies had to be accessible in English or French. Sub-national or provincial plans were excluded. We synthesised information on strategies’ considerations of inequity through a thematic analysis.

**Results:**

Of the 15 dementia strategies that met inclusion criteria, 13 mentioned at least one inequity (*M* = 2.4, *median* = 2, range:0–7) related to Race/Ethnicity; Religion; Age; Disability; Sexual Orientation/Gender Identity; Social Class; or Rurality. Age and disability were mentioned most frequently, and religion most infrequently. Eleven strategies included general inequity-focused objectives, while only 5 had specific inequity-focused objectives in the form of tangible percentage changes, deadlines, or allocated budgets for achieving equity-related goals outlined in their strategies.

**Conclusions:**

Understanding if and how countries consider inequities in their dementia strategies enables the development of future strategies that adequately target inequities of concern. While most of the strategies mentioned inequities, few included tangible objectives to reduce them. Countries must not only consider inequities at a surface-level; rather, they must put forth actionable objectives that intend to lessen the impact of inequities in the care of all persons living with dementia.

**Supplementary Information:**

The online version contains supplementary material available at 10.1186/s12939-024-02166-8.

## Background

The World Health Organisation (WHO) has designated dementia as a public health priority that requires urgent public health advocacy and global policies to mitigate its massive burden on the individual, community, economy, and healthcare system [1]. Dementia is a “syndrome of cognitive impairment that affects memory, cognitive abilities, and behaviour” [2] that hinders one’s ability to engage in activities of daily living and is a “major cause of disability and dependency” [3] primarily among older adults. The WHO set a global target for 75% of its Member States to develop or update national strategies for dementia by 2025 [3]. The WHO defines national dementia strategies as a set of principles and objectives written and published by a government authority for reducing the burden of dementia [4]: strategies commonly include multisectoral approaches to dementia care; accessible, affordable care that meets the needs of persons living with dementia and their families; raising awareness and eliminating stigma surrounding dementia diagnoses; and funding and support of research initiatives [5]. The WHO guidelines urge countries to select and apply recommended strategies and goals that take into account their different socioeconomic, political, and healthcare priorities [3]. Although the WHO encourages targeting inequities in dementia care, countries may develop national plans that do not meet the needs of the entire population, especially vulnerable groups that are more severely impacted by dementia.

While core elements of dementia are experienced by all persons who are affected, people living with dementia experience the disease differently based on their own lived experiences which are shaped by their social determinants of health (SDH) [6]. SDH are the conditions under which people are born, grow, live, work, and age, and encompass broad biological and social factors that shape health status and health outcomes, including economic stability, education, social and community contexts, health and healthcare, and neighbourhood and built environments [7, 8]. Inequalities exist across all facets of human life; however, when SDH give rise to systemic, avoidable, and unfair differences in outcomes and distribution of resources between groups, inequities arise [9]. Health inequities place populations who are already more vulnerable at an even greater disadvantage, by impacting their ability to access healthcare, receive appropriate health management, and engage with health systems [10]. Such inequities permeate all aspects of health and healthcare, and dementia is no exception–SDH not only affect the incidence, prevalence, and risk of dementia, but also play a role in determining disease progression and health outcomes [11–13]. SDH that have been widely researched and deemed to impact dementia risk and outcomes include race, gender, and socioeconomic status (SES): ethnic minorities have higher dementia incidence, greater cognitive decline, lower rates of diagnosis, and poorer uptake of medications [12, 14]; women are disproportionately impacted by dementia and have much higher diagnosis rates [15, 16]; and lower SES is associated with higher incidence, accelerated cognitive decline and more severe prognosis [12, 17, 18]. Minoritised individuals, such as racialised or queer people, consistently face barriers to accessing diagnostic, treatment, and support services for dementia, reflecting long-standing inequalities built into nations’ health systems [19].

To meet the WHO’s guidelines for developing dementia strategies that support and provide care for *all* citizens, it is crucial to consider inequities related to SDH in national dementia strategies: this consideration must be *broad* and cover a range of SDH that are known to affect dementia outcomes [1]. Beyond just considering what SDH are targeted, critically analysing the *depth* of objectives is important for understanding how thoroughly different countries engage with SDH in dementia care and how they plan to ameliorate inequities. This study aimed to describe the extent, or *scope*, of national dementia strategies in addressing inequities associated with SDH. By examining the depth, breadth, and scope of SDH in national dementia strategies, we aim to better understand trends in how inequities are considered in national-level dementia policy and enable the development of future strategies that adequately target inequities of concern.

## Methods

### Design and conceptual framework

To describe the inclusion of equity-related concerns in the national dementia strategies of countries, we conducted an environmental scan. Within the context of delivering health services, an environmental scan is a “type of inquiry that involves the collection and synthesis of existing information and/or the pursuit of new evidence to inform decision-making and help shape future response(s) to existing and emerging policy and service delivery issues and opportunities” [20]. This method not only allowed us to identify gaps in dementia strategies, but also aligned with our focus on ensuring equitable dementia care through policies and decision-making.

Our analysis was guided by two internationally recognised conceptual frameworks: (i) the United Nations’ System Shared Framework for Action on Equality and Non-Discrimination [21] and (ii) the WHO’s Conceptual Framework for Action on the Social Determinants of Health [22]. These frameworks are widely used to address inequalities and discrimination in the development and regulation of global institutions—we chose to merge both frameworks to consider all the SDH deemed relevant by the research team. Thus, we considered the following SDH in this study: Race/Ethnicity, Religion, Age, Disability (long-term physical, mental, intellectual, or sensory impairments [23]), Sexual Orientation/Gender Identity (including cisgender women), Social Class, and Rurality.

### Selection of dementia strategies

Dementia strategies from countries that are a part of the Organisation for Economic Co-operation and Development (OECD) were included to maximise comparability, since OECD countries have similar standards for economic development and corporate governance, and comparable healthcare conduct and principles. Only national strategies published in English or French were included; and for countries that published many dementia strategies over time, only the most recent version was included. Sub-national or province-specific strategies, and those that were not publicly available in the languages listed above, were excluded. We refer to all publications of dementia-related national policies as dementia strategies, considering that terms such as strategy, plan, policy, and framework are used interchangeably in this context [3].

### Search strategy

We consulted two websites and databases in December 2021 to find the current national dementia strategies of the 38 OECD countries, namely: (i) Alzheimer Europe’s website database of National Dementia Strategies [24], and (ii) Alzheimer’s Disease International’s list of national, sub-national, and non-governmental dementia strategies [25]. For the OECD countries that were not mentioned on either database, a secondary search was done to individually identify those countries’ dementia strategies using a standard Google Search.

### Data extraction and analysis

Based on our searches, we first identified countries which had a national strategy and determined whether to include them in this study based on our inclusion criteria. Then, to analyse whether the dementia strategies considered inequities and to what extent, we distinguished between three different stages of considering inequities: (1) mentioning SDH and inequities, (2) having general inequity-targeted objectives, and (3) having specific inequity-targeted objectives. A *mention* of SDH and inequities was defined as any brief naming or acknowledgment of an SDH or inequity. Having a *general inequity-targeted objective* was defined as a reference which summarised an overall intention or goal, without necessarily mentioning tangible end points, like quantified targets, timeframes, or specific budgets. A *specific inequity-targeted objective* was defined as a reference which was associated with either quantified target goals; or specific deadlines/year targets; or allocated budgets to achieve these specific goals.

To identify relevant data in each strategy, we searched the texts for each of the terms listed in Table 1 and extracted data and specific quotations pertaining to the aforementioned three stages. We organised the extracted data into the seven overarching categories of SDH, as per our conceptual frameworks. The following outcomes were considered and synthesised: (i) number of strategies mentioning each SDH; (ii) number of strategies that included general inequity-targeted objectives; and (iii) the number of dementia strategies that included detailed specific inequity-targeted objectives. Afterwards, we conducted a hybrid thematic content analysis and analysed the specific language, context, and area of focus to provide a consolidated, qualitative description of how the national dementia strategies of OECD countries mentioned Race/Ethnicity, Religion, Age, Disability, Sexual Orientation/Gender Identity, Social Class, and Rurality [26].

**Table 1:** Search Terms Used to Identify Relevant Data

English Terms	French Terms
Inequ*	Iniqu*/inégal*
Divers*	Diversif*/diversit*
Vulnerable	Vulnérable
Inclusi*	Inclusivement/inclusion
Cultur*	Culture/culturellement sûr
Objective*	Objectif
Recommend*	Recommand*
Target	Cible
Goal	
Program	Programme

## Results

### Search results, included strategies, and inequities mentioned

**Fig. 1:**
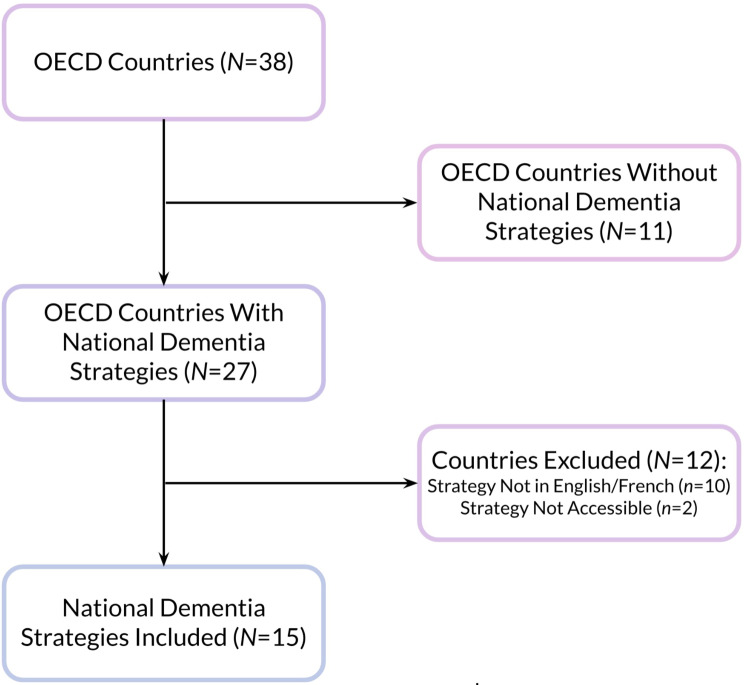
Flowchart of Included Dementia Strategies (adapted from PRISMA guidelines)

Of the 38 OECD countries, 27 had national dementia strategies. After removing dementia strategies based on the inclusion and exclusion criteria, a total of 15 dementia strategies were included (see Fig. 1; Table 2).

**Table 2:** OECD Countries and Their National Dementia Strategies

OECD Country	National Strategy?	Title and Date of Latest Strategy	Included in Study? If NO - reason
Australia	YES	National Framework of Action on Dementia (2015–2019) [27]	YES
Austria	YES	Dementia Strategy: Living well with dementia (2015) [28]	YES
Belgium	NO		NO– no strategy
Canada	YES	A Dementia Strategy of Canada: Together We Aspire (2019) [29]	YES
Chile	YES	Plan Nacional de Demencia (2017–2025) [30]	NO– Spanish
Colombia	NO		NO– no strategy
Costa Rica	YES	Plan Nacional Para la Enfermedad de Alzheimer Y Demencias Relacionadas Esfuerzos Compartidos (2014–2024) [31]	NO– Spanish
Czech Republic	YES	National Action Plan for Alzheimer’s Disease and related Illnesses (2020–2030) [32]	NO– Czech
Denmark	YES	A Safe and Dignified Life with Dementia: National Action Plan on Dementia (2025) [33]	YES
Estonia	NO		NO– no strategy
Finland	YES	National Memory Programme: Creating a “Memory Friendly” Finland (2012–2020) [34]	YES
France	YES	Plan Maladies Neuro-Dégénératives (2014–2019) [35]	YES
Germany	YES	National Dementia Strategy (2020) [36]	YES
Greece	YES	National Action Plan for Dementia– Alzheimer’s Disease (2016–2020) [37]	YES
Hungary	NO		NO– no strategy
Iceland	YES	Aðgerðaáætlun Um Þjónustu Við Einstaklinga Með Heilabilun (2020–2025) [38]	NO– Icelandic
Ireland	YES	The Irish National Dementia Strategy (2014) [39]	YES
Israel	YES	Addressing Alzheimer’s and Other Types of Dementia: Israeli National Strategy (2013) [40]	YES
Italy	YES	Piano Nazionale Demenze - Strategie per la Promozione ed il Miglioramento Della Qualità e dell’Appropriatezza Degli Interventi Assistenziali Nel Settore Delle Demenze (2014) [41]	NO– Italian
Japan	YES	New Orange Plan (2015) [42]	NO– not accessible
Korea	YES	The 3rd National Dementia Plan: Living well with dementia in the community (2015) [43]	YES
Latvia	NO		NO– no strategy
Lithuania	NO		NO– no strategy
Luxembourg	YES	Rapport Final du Comité de Pilotage en Vue de L’établissement d’un Plan D’action National «Maladies Démentielles» (2013) [44]	YES
Mexico	YES	Plan de Acción Alzheimer y Otras Demencias (2014) [45]	NO– Spanish
Netherlands	YES	National Dementia Strategy (2021–2030) [46]	YES
New Zealand	NO		NO– no strategy
Norway	YES	Dementia Plan (2025) [47]	NO– not accessible
Poland	NO		NO– no strategy
Portugal	YES	Estratégia da Saúde Na Área das Demências (2018) [48]	NO– Portuguese
Slovak Republic	NO		NO– no strategy
Slovenia	YES	Strategija Obvladovanja Demence v Sloveniji do Leta (2016–2020) [49]	NO– Slovenian
Spain	YES	Plan Integral de Alzheimer y Otras Demencias (2019–2023) [50]	NO– Spanish
Sweden	YES	Nationell Strategi för Omsorg om Personer med Demenssjukdom (2018) [51]	NO– Swedish
Switzerland	YES	Stratégie Nationale en Matière de Démence (2014–2019) [52]	YES
Turkey	NO		NO– no strategy
United Kingdom	NO		NO– no strategy
United States	YES	National Plan to Address Alzheimer’s Disease: 2018 Update [53]	YES

Of the 15 included dementia strategies, 13 mentioned at least one SDH in the context of inequities in dementia (see Table 3). The strategies ranged from mentioning 0 to 7 SDH, with an average of 2.4 and median of 2 SDH mentioned. Germany’s national strategy was the only one that mentioned all 7 SDH, whereas Luxembourg and Switzerland’s plans did not consider any SDH [36, 44, 52]. Of the types of SDH, age (60%) and disability (53%) were the most mentioned, whereas religion was only mentioned in one strategy (7%).

### How are inequities mentioned in dementia strategies?

**Fig. 2:**
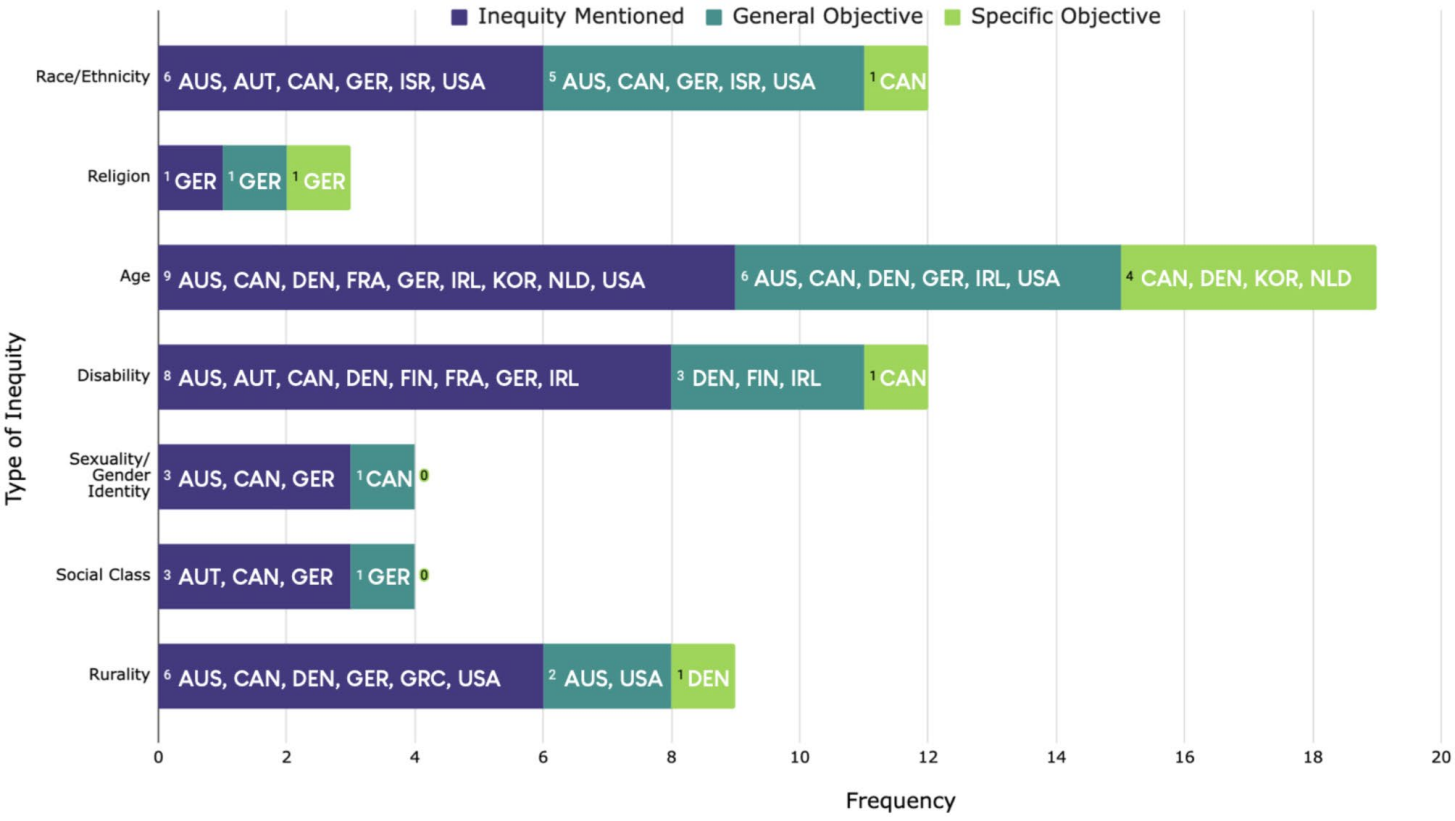
Frequency of Inequities Mentioned by Dementia Strategies by Country *Note* Countries are identified according to ISO 3166-1 alpha-3 codes used by the International Organization for Standardization (ISO) [54]

There is a large amount of variation in the type, amount, and depth of SDH and related inequities mentioned in dementia strategies. While some countries simply mentioned inequities related to SDH within a few sentences, others dedicated full chapters to inequities in dementia care. Similarly, while some countries only mentioned general objectives to target inequities, others detailed specific targeted objectives to address them. Figure 2 provides an overview of which national strategies mentioned and established general and/or specific objectives to mitigate each inequity. We explore this heterogeneity more in-depth.

### Race/ethnicity

**Mentions of Race/Ethnicity.** Six dementia strategies mentioned race and/or ethnicity [27–29, 36, 40, 53]. Race and ethnicity are described not only as barriers to seeking medical care, but also as barriers to receiving medical care. For example, certain dementia plans reported on specific populations that face stigma and may hold negative perceptions about dementia care. These populations avoid seeking out dementia care for fear of it being considered taboo or ‘not a medical condition,’ leading individuals in these communities reluctant to seek out or accept support [27]. Alternatively, some dementia strategies focused primarily on equitable access to dementia care, and foregrounded targets that could be pursued to mitigate barriers to accessing care. For example, many racialised groups that actively seek out dementia care are met with barriers regarding communication or culturally appropriate care. Strategies placed strong emphasis on the need to improve service delivery by collaborating with people of minority or migrant backgrounds.

**General Objectives That Target Inequities Related to Race/Ethnicity.** Five dementia strategies mentioned general objectives to target race- and/or ethnicity-related inequities [27, 29, 36, 40, 53]. Generally, the main focus of these objectives was the development of culturally appropriate and safe care and informative resources, especially when developed through collaboration with Indigenous groups in Canada and Australia [27, 29]. Some of these objectives include improving support for culturally sensitive counselling, developing culturally safe guidelines for standards of care, and developing culturally sensitive education, support, and training [27, 29, 36, 53]. Another focus of the general objectives is increasing the involvement of racial and ethnic minorities in research, with goals to both increase enrolment of racial and ethnic minorities and including minority groups at all stages of research [40, 53].

**Specific Objectives That Target Inequities Related to Race/Ethnicity.** One strategy included a specific budget for the overall plan but did not specify resource allocation towards racial and ethnic minorities [29].

### Religion

**Mentions of Religion.** One of the 15 dementia strategies mentioned religion and/or religious values [36]. The spiritual and religious needs of persons living with dementia were reported as a target of the national dementia strategy, placing emphasis on the need for support tailored to one’s own life history and religious faith, both to ease the burden of dementia and to improve counselling and education on dementia.

**General Objectives That Target Inequities Related to Religion.** The same dementia strategy also included a general objective that targeted inequities related to religion. This objective aimed to support the spiritual and religious needs of persons living with dementia [36].

**Specific Objectives That Target Inequities Related to Religion.** This strategy also included specific deadlines to meet religion-related targets: namely, providing culture- and religion-sensitive support and counselling services, and incorporating pastoral workers and care into local counselling structures [36].

### Age

**Mentions of Age.** Nine of the 15 dementia strategies mentioned age [27, 29, 33, 35, 36, 39, 43, 46, 53]. Generally, age as a risk factor for inequitable health care was directed at early-onset dementia, which occurs when dementia onset occurs before the age of 65, as is often the case with frontotemporal dementia [55, 56]. Early-onset dementia was predominantly described as a barrier to accessing appropriate services, given that dementia services and programs tend to be designed around the interests and needs of older populations [27, 29, 39]. An emphasis was placed on the need for age-appropriate services that preserve the quality of life for younger people living with dementia.

Alternatively, old age was also mentioned as a barrier to dementia care, specifically looking at the management of higher risk groups, groups with co-morbidities, or those who are more prone to worse outcomes of dementia [35, 43].

**General Objectives That Target Inequities Related to Age.** Six dementia strategies included age-related general objectives that can be organised into two categories: support for early-onset dementia, and support for older persons living with dementia [27, 29, 33, 36, 39, 53]. Five out of the six dementia strategies focused on support for early-onset dementia – the main focus of these objectives were extending support and counselling services for younger persons living with dementia and their families, developing care pathways and home services that are flexible for those with early-onset dementia, and increasing support for younger people with dementia to remain in their employment and offer support for child services [27, 33, 36, 39, 53].

One strategy specifically mentioned objectives related to older people with dementia and sought to support communities that had taken steps to be age-friendly and inclusive of older people with dementia [29].

**Specific Objectives That Target Inequities Related to Age.** One strategy included a specific budget for the overall plan, including early diagnosis, but did not specify resource allocation towards certain age brackets [29], whereas another included allocating resources to developing tools for early dementia detection and establishing counselling and activity centres for people with dementia and their care partners, with a focus on younger people with dementia [33]. One specified a budget for developing ageing-friendly products to support independent living in older adults [43]. Another strategy established a deadline for care centres and municipalities to acquire adequate insight into the residential needs of older people with dementia [46].

### Disability

**Mentions of Disability.** Eight of the 15 dementia strategies mentioned disability [27–29, 33–36, 39]. The definition of disability among the strategies greatly varied. Six of these strategies focused on persons with intellectual disabilities: based on the content of the dementia strategies, intellectual disability was defined as comorbid cognitive impairments beyond those that would accompany a diagnosis of dementia, including persons who are also diagnosed with Down syndrome or another diagnosed disability. There are 2 main focuses of targeted initiatives for persons with intellectual disabilities: decreasing the stigma surrounding disabilities that may lead to discrimination against and exclusion of persons living with dementia from treatment or to the refusal of appropriate treatment; and communicating research and providing opportunities in ways that increase accessibility and are culturally appropriate [29, 33].

Another strategy briefly mentions “people with physical disabilities” as a population that is increasingly becoming vulnerable to the development of late-onset dementia [27].

**General Objectives That Target Inequities Related to Disability.** Three strategies included objectives that focused on including persons living with dementia who also have intellectual or physical disabilities at all stages of care and care planning, including taking legislative action [33, 34, 39]. Specific examples of this include taking people with disabilities into account when planning activities that promote brain health, deploying resources that are accessible to those with intellectual disabilities, and working on reliable diagnostic tests for persons with young-onset dementia who also have intellectual disabilities [34, 39].

**Specific Objectives That Target Inequities Related to Disability.** One strategy included an allocated budget for improved home and community care but did not mention specific disabilities or conditions [29].

### Sexual orientation/gender identity

**Mentions of Sexual Orientation/Gender Identity.** Three of the 15 dementia strategies mentioned sexual orientation and/or gender identity [27, 29, 36]. Two mentioned specific support relating to those who identify as lesbian, gay, bisexual, transgender, intersex, and queer (LGBTIQ+), focusing on services that are sensitive to the needs of these communities [27, 29]. One of these needs is providing support to vulnerable populations, specifically those that have difficulties accessing diagnoses and care due to potential stigma and social marginalisation [29]. Difficulties include factors like trust and disclosure of sexual orientation, fear of being mistreated, and discrimination in long-term care homes.

Two strategies listed women, specifically older women, as a group that requires specific support for their status as ‘at-risk’ or more vulnerable to developing dementia [29, 36].

**General Objectives That Target Inequities Related to Sexual Orientation/Gender Identity.** One strategy aimed to prioritise dementia-care related projects that specifically targeted women as a vulnerable population [29]. There were no objectives related to non-binary identities or sexual orientation.

**Specific Objectives That Target Inequities Related to Sexual Orientation/Gender Identity.** None of the strategies included specific objectives related to sexual orientation and gender identity.

### Social class

**Mentions of Social Class.** Three of the 15 dementia strategies referenced social class as an area of concern [28, 29, 36]. Two referred to social class-related inequalities in access to dementia care [29, 36]. Specific concerns included a focus on access to help for those experiencing homelessness, and supporting access to care for people living with dementia and their caregivers who may face socio-economic marginalisation [28, 29].

**General Objectives That Target Inequities Related to Social Class.** One strategy aimed to conduct research that considers social and socioeconomic inequalities as factors that are relevant to the development of dementia and to the treatment and care of persons living with dementia [36].

**Specific Objectives That Target Inequities Related to Social Class.** None of the strategies included specific objectives to target the impact of social class on dementia care.

### Rurality

**Mentions of Rurality.** Six of the 15 dementia strategies mentioned rurality [27, 29, 33, 36, 37, 53]. All of these strategies present rurality as a barrier to accessing dementia care and related resources. Emphasis was placed on the lack of specialists and multi-disciplinary teams in rural and remote communities [27, 37, 53]. Responses to this include supporting regional, rural, and remote communities by ensuring that all municipalities are dementia-friendly with counselling services that are easily located, and by focusing on rural development of “dementia-sensitive” social spaces and accessible transportation to rural areas [29, 33, 36].

**General Objectives That Target Inequities Related to Rurality.** Two strategies had general objectives that focused on ensuring that resources were made available to rural or remote locations [27, 53]. This included providing services and adequate response to support rural and remote communities, specifically by increasing accessibility to primary and specialist care services and distributing new resources to rural areas and Indigenous communities [27, 53].

**Specific Objectives That Target Inequities Related to Rurality.** One strategy included a specific deadline for reducing the geographical inequality between municipalities and regions within the country [33].

**Table 3:** Types of Inequities Mentioned by Dementia Strategies by Country

Country	Inequities Mentioned?	Type of SDH Mentioned								General Objective Mentioned?	Specific Objective(s) Mentioned?		
		Race & Ethnicity	Religion	Age	Disability	Sexuality & Gender	Social Class	Rurality	N		Target: Quantified Goal	Target: Specific Deadline	Budget or Financial Allocation
Australia	YES	✓		✓	✓	✓		✓	5	YES			
Austria	YES	✓			✓		✓		3	NO			
Canada	YES	✓		✓	✓	✓	✓	✓	6	YES			✓
Denmark	YES			✓	✓			✓	3	YES		✓	✓
Finland	YES				✓				1	YES			
France	YES			✓	✓				2	NO			
Germany	YES	✓	✓	✓	✓	✓	✓	✓	7	YES		✓	✓
Greece	YES							✓	1	YES			
Ireland	YES			✓	✓				2	YES			
Israel	YES	✓							1	YES			
Korea	YES			✓					1	YES			✓
Luxembourg	NO								0	NO			
Netherlands	YES			✓					1	YES		✓	
Switzerland	NO								0	NO			
United States	YES	✓		✓				✓	3	YES			
Total: N (%)	15	6 (40)	1 (7)	9 (60)	8 (53)	3 (20)	3 (20)	6 (40)					

## Discussion

Our environmental scan found that most OECD countries have national dementia strategies, and that almost all the included strategies mentioned at least one SDH and discussed broader general inequity-targeted goals. However, only a third of the national dementia strategies included tangible targets to mitigate these inequities, in the form of either specific deadlines, quantified targets, or allocated budgets. Strategies were predominantly geared towards people with dementia, though some discussed decreasing missed dementia diagnoses, improving diagnostic services, and working with underserved communities to develop culturally appropriate diagnostic tools [27, 29, 36].

Considering that there are inequities in dementia risk, outcomes and care, national dementia strategies can be powerful instruments to help mitigate SDH-related inequities, especially by providing specific objectives to target inequities that are especially relevant to dementia care provided to their populations [12, 14–18]. Furthermore, as countries embrace strategies to tackle inequities in their dementia plans, this effect could snowball and inspire other nations to incorporate SDH-addressing strategies in their own plans.

Our findings echo those of a 2022 systematic review investigating protection against discrimination in national guidelines for assessment, diagnosis, and management of dementia [57]. The authors highlighted that although most guidelines mentioned SDH and inequities, only a fraction included specific recommendations to mitigate them [57]. The authors did not specifically gauge whether or not these recommendations were associated with tangible objectives, whereas our study investigates how deeply strategies considered inequities, that is, the inclusion of broad and specific mitigating objectives.

### Why target inequities?

For strategies and interventions to effectively reduce inequities, they must not only target the population at large, but also target specific, vulnerable populations [58]. Implementing population-level national plans without taking into consideration how the strategy may differentially impact communities within the population runs the risk of inadvertently increasing inequities, since the least marginalised will have greater access and better outcomes, thereby widening the gap across SDH [58]. Interventions must shift the risk exposure distribution of vulnerable groups while also targeting large-scale social and environmental conditions that shape how groups experience risk—such an approach assures that health risk is lowered across all populations, without increasing inequities and creating further divides between groups [58]. Our study found that, on the contrary, most countries primarily targeted the general population with dementia in their goals–while a good first step, this approach is at risk of lowering the ‘average’ risk while increasing the range in risk distribution, causing those that were originally most vulnerable to face even greater risk of adverse outcomes [58]. Countries like Sweden and New Zealand have implemented policies (in areas other than dementia) specifically targeting social inequities in their public health policies, which have led to tangible results: health improved both in vulnerable as well as general populations [59]. These experiences suggest that by implementing policies focused on those who are most vulnerable, the overall health of a population improves. Therefore, by targeting inequities in national dementia strategies, not only will policy makers be tackling significant disparities in healthcare, but also promoting better health for non-minoritised and minoritised people with dementia. Using this approach could be instrumental in improving overall health, care and service use, and quality of life of persons with dementia, and may lead to better health for all rather than excellent health for some.

### Considering inequities: from mention to action

It is therefore promising that OECD countries do seem to take into account the issue of inequities in dementia care, given that most of the included countries’ strategies mentioned at least one SDH and a general objective to target an inequity. However, our scan showed that mentioning SDH is not equivalent to specifying objectives with actionable targets to mitigate inequities. As we have shown, there are discrepancies and substantial variation in *how many* SDH and inequity-targets countries’ plans mention, *which* SDH are mentioned or targeted, and *how much* the inequities and targets are considered. Few strategies actually considered inequities in depth. While mentioning SDH and inequities in the context of dementia is a good starting point, this ‘surface-level’ approach to tackling inequities might not suffice and is unlikely to ensure tangible changes throughout the health care system to mitigate inequities in dementia—implementing such changes might require more specific targets.

For strategies to be accurately employed in healthcare settings and communicated clearly to a variety of stakeholders, they must be specific, not just conceptually but also operationally [60]. Specificity also ensures that the targets and goals that are set out in the creation of strategies are also enacted in the intended manner [60]. Therefore, if countries intend to address disparities in dementia care, it is crucial that their national dementia strategies include actionable, specific objectives.

### Strengths and limitations

First, this scan was limited to national dementia strategies and did not include all available documentation related to the implementation of these strategies. Given that national dementia strategies could be limited in what and how much they report, it is possible that information on tangible goals to mitigate inequities was made available elsewhere and therefore inadvertently left out of this scan. Further research that looks at all available documentation, such as by contacting relevant governmental agencies, may be necessary to confirm our results. Second, this environmental scan focused only on national-level policies and excluded all subnational-level dementia strategies. Although this focus allowed for the most inter-country comparable analysis of dementia strategies, it is possible that sub-national strategies could have addressed more specific inequities as they pertain to smaller cohorts of a population. The focus on national-level strategies also excludes some countries, for example, the United Kingdom, where the most recent strategies were sub-national. We also only included strategies in English and French: therefore, we could not review ten strategies, from countries such as Mexico and Portugal, that altogether represent over 293 million people [61]. Chile’s national strategy, for instance, addresses each of the action areas and indicators set forth by the WHO Global Action Plan and the Pan American Health Organisation, respectively [62]. By excluding strategies based on language, we were unable to assess whether such thorough strategies considered SDH in their dementia plans. Furthermore, for greater inter-strategy comparability, we focused on OECD countries: our overview is limited to high-income countries and does not capture the dementia-care priorities of low- and middle-income countries. Further research could benefit from less stringent inclusion criteria to better capture SDH considerations across the world.

Despite these limitations, this environmental scan presents a thorough synthesis of OECD countries’ national dementia strategies and objectives to mitigate inequities in dementia care. Other noteworthy strengths include examining current dementia policy through an equity-focused lens, which fills an important gap in dementia policy research that is necessary to inform future policy in a way that specifically targets vulnerable populations to bridge healthcare gaps. Furthermore, this scan synthesised in detail how policies considered SDH and inequities, from a general to a specific definition: quantifying the level to which national strategies engaged with equitable care allows for a thorough understanding of the depth and scope of dementia strategies’ objectives to mitigate inequities in dementia care. Additionally, our consideration of multiple SDH, rather than fixating on a singular type of SDH, offers insight into how countries approach the different disparities that affect dementia outcomes and care. For instance, although it is well established that lower socioeconomic status is associated with poorer outcomes and care in dementia, none of the strategies included specific goals targeting social class [12, 17, 18].

### Recommendations

We recommend that future work not only expand current knowledge by assessing the state of SDH outside OECD countries and across languages, but also that it evaluate whether countries enact their outlined general and specific objectives. Countries themselves should be motivated to summarise the existing state of affairs and make available data on policies they were and were not able to undertake. In addition, justifying the inclusion or exclusion of various SDH and equity-related concerns within national dementia strategies can make countries’ priorities clearer. Nations can also clarify how dementia strategies are situated within broader national health policies, which may illuminate whether upper-level considerations of equity trickle down into dementia policy, without inequities being explicitly included in national plans themselves.

When developing national dementia strategies, healthcare systems should incorporate perspectives from experts across fields, such as social economists, to build viable public policies that address each country’s socio-political landscape. As the number of people with dementia rises, especially in low- and middle-income countries, it is imperative to establish greater international collaboration so that countries build better policies by not building their strategies in isolation [63]. Instead, nations can support one another in reducing the immense social, economic, and individual costs inherent to dementia, and ensuring that lower-income countries do not bear disproportionate burdens of the disease.

## Conclusion

This study presents an international and current synthesis of considerations of inequities in national dementia policy. This scan showed that almost all the included dementia strategies had at least mentioned SDH and general inequity-targeted goals, but few had objectives to mitigate SDH-related inequities with tangible targets, a step that might be necessary to actually tackle inequities. Based on the results of this scan, and using existing frameworks to support them, when developing future dementia strategies, countries could identify the inequities of concern in their specific populations, adopt both general-population and vulnerable-population approaches to health interventions, and then frame specific, quantifiable, timely, and budgeted objectives towards mitigating inequities in health and healthcare. This might help mitigate the current inequities in dementia care and ensure the best quality of care for *all* people with dementia.

### Electronic supplementary material

Below is the link to the electronic supplementary material.

Supplementary Material 1

## Data Availability

No datasets were generated or analysed during the current study.
