# Supplementary material for: Considering inequities in national dementia strategies: breadth, depth, and scope
Source: Int J Equity Health. 2024 Apr 16;23:75. doi: 10.1186/s12939-024-02166-8 (PMC11022480; doi:10.1186/s12939-024-02166-8)
Supplement: Supplementary file 1 — Supplementary Material 1 [file 12939_2024_2166_MOESM1_ESM.docx]

**Appendix**

**Table 1.** Quotations from Countries’ National Dementia Strategies, Concerning Mentions of Social Determinants of Health (SDH)

| **Country** | **SDH** | **Mentions** | **General Objectives** | **Specific Objectives** | | |
| --- | --- | --- | --- | --- | --- | --- |
|  |  |  |  | Target Percent | Target Year/Deadline | Allocated Budget |
| Australia | Race/Ethnicity | Lack of awareness and access to services for Aboriginal and Torres Strait Islander people - studies indicate that the prevalence of dementia in the Aboriginal and Torres Strait Islander populations is over five times greater than the general population. While prevalence of dementia is likely to be greater in Aboriginal and Torres Strait Islander communities, awareness of dementia in these communities is lower than in the overall Australian population.  Also, the perception of dementia can be quite different across Aboriginal and Torres Strait Islander communities. It is often not viewed as a medical condition, and consequently medical treatment or support is not often sought.  Different cultural perceptions of dementia are present in culturally and linguistically diverse (CALD) communities. In some communities, dementia is a taboo issue which is not openly discussed, resulting in even higher levels of stigma and negative community perceptions. These cultural perceptions impact on individual willingness to access services and decrease the likelihood of accepting support. | Tailored early support services needed for those who are in diverse populations  Support culturally appropriate care for people with dementia from diverse needs groups (Aboriginal and Torres Strait Islander, CALD communities)  Enhance quality and availability of services for diverse needs groups through improved education and training for the workforce |  |  |  |
|  | Age | Younger Australians with dementia may face many challenges similar to those of older people with dementia, however the non-normative timing of the disease and different practical considerations present challenges different to those faced by older people with dementia.  Younger people may face barriers to accessing appropriate services as dementia services tend to be designed around the interests and physical abilities of older people. | Develop clinical referral and care pathways that are flexible including for people with dementia from diverse needs groups and those with younger onset dementia  Provide support for people with younger onset dementia to remain in employment for as long as possible and maintain family/community participation  Provide age appropriate home, residential, and acute care support services for people with younger onset dementia, their carers, and families  Develop clinical referral and care pathways that are flexible including for people with dementia from diverse needs groups and people with younger onset dementia |  |  |  |
|  | Disability | People with physical and intellectual disabilities are increasingly surviving to older age and therefore are vulnerable to age- associated disorders such as dementia. It is reported that one in five people, with an intellectual disability, aged 65 and older, have dementia  People with disability who are vulnerable to age- associated disorders |  |  |  |  |
|  | Sexual Orientation/Gender Identity | Those people who identify as Lesbian, Gay, Bisexual, Transgender and Intersex (LGBTI) require specific supports and services that are sensitive and respectful to their needs |  |  |  |  |
|  | Rurality | People living in rural and remote communities face many challenges including access to primary and specialist health care. There may be a shortage of specialists and established multi- disciplinary teams, particularly in regional, rural and remote communities of Australia. In these locations, distance often presents a significant barrier to accessing primary and specialist care services. | There is a requirement for service responses to support regional, rural and remote communities of Australia. |  |  |  |
| Austria | Race/Ethnicity | Focus on the equality of access to offers for help for minorities and people with migrant backgrounds |  |  |  |  |
|  | Disability | Focus on the equality of access to offers for help for people with disabilities |  |  |  |  |
|  | Social Class | Focus on the equality of access to offers for help for those who are homeless |  |  |  |  |
| Canada | Race/Ethnicity | Research findings are communicated in ways that increase accessibility and are culturally appropriate across diverse communities such as Indigenous peoples, immigrant and minority language communities  For those who live on reserve, lack of access to health professionals and services on reserve, especially in rural and remote communities, as well as a lack of cultural safety in the health system create barriers to equitable care.  Cultural differences in the understanding of and views toward dementia can result in a reluctance to talk about symptoms and may lead to under- diagnosis and difficulty connecting with networks of support that are useful following diagnosis. Understanding the impacts of dementia among ethnic minority populations is limited in Canada | Developing and sharing evidence- informed services, information and resources related to dementia care and advance care planning, and adapting them to different cultures, populations and languages  Work collaboratively with Indigenous communities to develop culturally safe and culturally appropriate tools for diagnosis.  Improved access to evidence- informed, culturally safe and culturally appropriate guidelines for standards of care |  |  | Budget 2019 provides $50 million over 5 years, starting in 2019-20, to support the implementation of the national dementia strategy and to work with key stakeholders to: increase awareness about dementia through targeted campaigns and activities that focus on prevention, reducing risk and stigma |
|  | Age | A lack of awareness of young onset dementia can lead to delays in diagnosis which result in delays in accessing needed supports and treatments. A lack of age- appropriate services is a significant barrier to the quality of life for people living with young onset dementia and caregivers. | Support in the form of a provincial recognition program for communities that have taken steps to be age-friendly and inclusive of seniors |  |  | Budget 2019 provides $50 million over 5 years, starting in 2019-20, to support the implementation of the national dementia strategy and to work with key stakeholders to develop treatment guidelines and best practices for early diagnosis |
|  | Disability | Research findings are communicated in ways that increase accessibility and are culturally appropriate across diverse communities such as people with intellectual disabilities  Adults with intellectual disabilities have experienced stigma, discrimination and exclusion that can continue following a dementia diagnosis. Those with intellectual disabilities may also have unique care and support needs, arising from the combination of their previous disability with the overlay of dementia symptoms. |  |  |  | In 2017, the Government of Canada provided provinces and territories with an additional $11 billion over 10 years specifically targeted to improve home and community care, including palliative care, and mental health and addiction services. (5 billion for mental health) |
|  | Sexual Orientation/Gender Identity | Research findings communicated in ways that increase accessibility and are culturally appropriate across LGBTQ2 communities  Strategy fills gaps in programs and supports for at-risk and vulnerable populations, including senior women  Some evidence indicates that LGB adults may experience delays in dementia diagnosis and difficulties finding supports due to stigma and social marginalization.  Transgender people face additional barriers to health services with a survey showing that 23% did not see a doctor or seek medical care for fear of being mistreated. Concerns have been raised by LGBTQ2 adults about possible stigma and discrimination in assisted living and long-term care homes as well as the challenges around trust and disclosure of sexual orientation | The Dementia Community Investment will prioritize projects targeting various populations, such as women |  |  |  |
|  | Social Class | Ethnic minority people living with dementia and caregivers face difficulties in accessing care and support due to socio- economic marginalization |  |  |  |  |
|  | Rurality | Research findings are communicated in ways that increase accessibility and are culturally appropriate across diverse communities such as those who live in Rural and remote communities  Development of these resources must be done in ways that ensure accessibility across cultures, languages and different geographic areas (such as rural/remote communities)  Health and social supports are typically more sparse in rural and remote regions compared with urban communities, which can result in people living with dementia having to leave their communities or travel long distances for care and supports. |  |  |  |  |
| Denmark | Age | A timely and accurate diagnosis is crucial in order to enable the municipalities and regions to provide an appropriate treatment and a qualified care for people with dementia. This is especially the case for the group of younger people affected by dementia, who are often even more difficult to diagnose | Increase in the number of places offered in relief day care centres and more support for younger people with dementia  Counselling- and activity centres for people with dementia and their relatives, including younger people with dementia |  |  | DKK 1.6 million is allocated to elaborate a tool – based on already existing tools - that can help to detect dementia at an earlier stage.  DKK 37.5 million is allocated to establish counselling- and activity centres for people with dementia and their relatives, focusing on younger people with dementia |
|  | Disability | Several citizens with a permanently reduced mental capacity, do not receive the somatic treatment they need to maintain good health because they refuse to receive treatment | The government and the political parties behind this action plan agree to continue the work on a new legislation that can strengthen the care for this group of citizens, so treatment will be possible despite their refusal. |  |  |  |
|  | Rurality | All 98 municupalities in Denmark should be dementia friendly - It is also important to ensure that information on the municipal efforts on dementia is easy accessible for people affected by dementia and their relatives and that counselling assistance is easily located |  |  | Three national goals for the dementia efforts towards 2025 have been set in order to contribute to a significant boost of the field of dementia and to diminish the geographical inequality between municipalities and regions. |  |
| Finland | Disability | Special attention must be given to the needs of vulnerable groups, such as people undergoing rehabilitation for mental health issues | Provide... residents with opportunities to take part in activities that promote brain health and for taking the needs of different kinds of people into account when planning such activities. |  |  |  |
| France | Age | Poursuivre les efforts en matière d'amélioration de la solvabilisation pour réduire les inégalités sociales qui s'aggravent avec l'âge ou la maladie et à faire rentrer la politique de l'autonomie dans l'ère du numérique |  |  |  |  |
|  | Disability | L'enjeu de qualité repose sur l'accès à une médecine de premier recours de qualité et à une organisation des soins spécialisés bien oordonnés. L'ensemble des professions de santé est concerné pour répondre à des besoins diversifiés : troubles moteurs, de l'équilibre, troubles du comportement, de la cognition, etc., conséquences de la maladie ou de son traitement.  Proposer des réponses adaptées à la diversité des groupes de personnes (demi- journées dédiées à la préservation des capacités motrices, à la préservation des capacités cognitives, etc.) |  |  |  |  |
| Germany | Race/Ethnicity | "Migration background inequality”  Intercultural competence at care counselling centers; further development and networking of culturally sensitive information services and counselling  Improving multilingual, culturally sensitive assessment instrument for diagnosing dementia | Field of action - developing and expanding culturally sensitive counselling services for people with dementia and their relatives |  |  |  |
|  | Religion | Religious people need support tailored to their own life history, even in old age. Knowledge about dementia, social integration and religious faith can ease the burden of the condition. More culture- and religion-sensitive support and education is therefore needed. | Supporting the spiritual and religious needs of people with dementia is one objective of the National Dementia Strategy. |  | By the end of 2024, culture- and religion- sensitive support and counselling services for family caregivers will be available.  By the end of 2024, employees in migrant support organisations will be trained on the topic of dementia.  By the end of 2022, the pastoral care for older people, offered by the evangelical and catholic churches in Germany, will be networked with other local counselling structures, and further pastoral workers for people with dementia will be trained. |  |
|  | Age | “People with early-onset dementia and their families” | Extending counselling and support structures for people with early-onset dementia and their families |  |  |  |
|  | Disability | The Inclusive Social Environments Initiative (ISI) of the BMAS, in coordination with the local authority associations, likewise aims to further improve the circumstances of people with disabilities. |  |  |  |  |
|  | Sexual Orientation/Gender Identity | The review will also consider the situation of foreign assistance and care staff, care provided by relatives living remotely, and issues relating to social inequality such as gender |  |  |  |  |
|  | Social Class | The quality of health services for people with dementia is also affected by social inequality. This, as well as the effects of the condition on everyday life, of people with dementia, will be considered. The quality of healthcare for people with dementia is influenced, amongst others, by psycho-social factors and social and socio- economic inequalities. For instance, a low level of education is associated, on average, with higher risks of more serious somatic disorders, including certain forms of dementia. These relationships are to be researched in greater depth. | Research will consider various factors relevant to the development of dementia and the treatment and care of people with dementia: psychosocial factors, social inequality, socioeconomic inequality and demographic patterns of ageing |  |  |  |
|  | Rurality | Focus on rural development of dementia- sensitive social spaces, and an increase in accessible transportation in rural areas |  |  |  |  |
| Greece | Rurality | Geographical inequalities of the existing services, which are gathered in a few large cities.  Moreover, the distribution of these services and structures is uneven and not spatially correct. Major shortages are in rural areas and in the islands |  |  |  |  |
| Ireland | Age | While dementia is associated with increasing age and usually begins to present in the population after the age of 65, people in their 30s, 40s or 50s can experience it. The diagnosis of younger onset dementia is challenging, with symptoms often confused with other disorders and disabilities, such as depression and other mental health problems. People with younger onset dementia are most commonly affected by Alzheimer’s Disease, Vascular Dementia and Dementia with Lewy Bodies. Some develop younger onset dementia alongside other disorders such as Down Syndrome, Parkinson’s disease, Acquired Immune Deficiency Syndrome (AIDS), Huntington’s disease, Creutzfeldt- Jakob disease, and alcoholism. The difficulties experienced by younger people with dementia are compounded by the fact that many are still employed in the labour market and will have financial responsibilities including mortgages. Many will also have parental and family responsibilities. People with early onset dementia usually experience greater difficulty accessing a diagnosis and fitting into existing dementia service provision, which is generally tailored to the needs of older people.  This Strategy addresses the needs of all people with dementia, including those with younger onset dementia. | Available resources should be deployed on the basis of need and as effectively as possible to provide services for all people living with dementia, including those with early onset dementia |  |  |  |
|  | Disability | "those with...an intellectual disability"  "target populations particularly at risk, including people with an intellectual disability" | The Health Service Executive will examine the issues arising regarding the assessment of those with Down Syndrome and other types of intellectual disability given the early age of onset of dementia for these groups and the value of establishing a reliable baseline  Available resources should be deployed on the basis of need and as effectively as possible to provide services for all people living with dementia, including those with... an intellectual disability, and should be delivered in a culturally appropriate way  Priority Action: target populations particularly at risk, including people with an intellectual disability |  |  |  |
| Israel | Race/Ethnicity | Ensuring the inclusion of minority groups and other sub- groups in the research agenda"  Developing a national program to disseminate information on dementia that is culturally adapted to Israeli society and to different cultural groups | Research includes minority groups and other sub- groups in research agenda |  |  |  |
| Korea | Age | Management of high risk group: Older adults 75+ living alone  Support for early diagnosis of dementia |  |  |  | Utilize the MHW ‘Coping with the ageing’ research development project (budget of 1.5 billion KRW) to develop aging-friendly products, and to support independent living of older adults |
| Netherlands | Age | Young persons with dementia: A special group within this strategy consists of young persons with dementia. They often have other forms of dementia than persons 65 years and older. In part due to their age, diagnosing dementia under these younger persons is more difficult as dementia is often overlooked in the first instance. As these persons often have a family with children still living at home, are physically stronger, and are still part of the labour force, their needs in terms of support and care are different |  |  | No later than the summer of 2021, municipalities and care centres have acquired sufficient insight into the residential needs of older persons and other target groups |  |
| United States | Race/Ethnicity | NIH is supporting the Reasons for Geographic and Racial Differences in Stroke (REGARDS) study and the Northern Manhattan Study (NOMAS) which are diverse longitudinal cohort studies of African American or Black, and Hispanic or Latino participants.  Development of culturally-competent dementia care specialists, dementia friendly community education/awareness initiatives and translation of the Music and Memory intervention in Indian Country  Main goal: Decrease disparities in Alzheimer's disease for racial and ethnic minority populations that are at higher risk for Alzheimer's disease | Monitor and identify strategies to increase enrollment of racial and ethnic minorities in Alzheimer’s disease and related dementias studies  Evaluate recruitment strategies for American Indians and Alaska Natives, create culturally tailored materials on AD-PM  Ensure receipt of culturally sensitive education, training, and support materials  Connect American Indian and Alaska Natives to Alzheimer's disease and related dementias resources  Development of culturally- competent dementia care specialists |  |  |  |
|  | Age | The population with younger-onset AD/ADRD faces unique challenges with diagnosis, care, and stigma. | HHS will undertake the actions below to better understand the unique challenges faced by these groups and create a plan for improving the care that they receive, which will be integrated into the broader efforts to improve care for all people with AD/ADRD.  Issue recommendations about standards for evaluating the effectiveness of treatments for early-stage AD/ADRD. |  |  |  |
|  | Rurality | Private interdisciplinary team training in recognition, assessment, and management of Alzheimer’s disease and related dementias in small rural Indian Health facilities | As new resources become available, they will be distributed through a variety of venues to Indian Country |  |  |  |
